# Quantification of safe operation conditions for large-area platinum-iridium electrodes in neurostimulation application

**DOI:** 10.1371/journal.pone.0315779

**Published:** 2024-12-19

**Authors:** Elisa Leusmann, Ciamak Abkai, Michael Tittelbach, Wigand Poppendieck

**Affiliations:** 1 University of Applied Sciences Mannheim, Mannheim, Germany; 2 Precisis GmbH, Heidelberg, Germany; Hamadan University of Medical Sciences, ISLAMIC REPUBLIC OF IRAN

## Abstract

**Objective:**

Using electrochemical characterization methods of stimulation electrodes as well as accelerated stimulation examinations, a safe operating field for stimulation is investigated for particularly very large Pt-Ir macroelectrodes in a Laplace configuration.

**Approach:**

Traditional methods such as Electrochemical Impedance Spectroscopy, Cyclic Voltammetry and biphasic, charge balanced current pulses were applied on Pt-Ir macroelectrodes in phosphate buffered saline solution to investigate reversible boundaries. These experiments were adapted to approach realistic working conditions.

**Main results:**

Investigating operational conditions close to realistic use cases have shown an anti-correlation between higher scan rates and the occurrence of irreversible reactions. In addition, at higher current pulse amplitudes (>3 *mA*), the voltage dropping across the phase boundary (*E*_*ma*_ and *E*_*mc*_) saturated. The value of the residual voltage depends on the degree of charge balance of the biphasic pulse. Thus, we have been able to detect more dissolved platinum at high residual voltages—objected by mass spectroscopic measurement.

**Significance:**

Residual voltage plays a greater role concerning reversibility in prolonged stimulation and charge imbalance of applied biphasic current pulses. The *E*_*ma*_ saturation is suggested as a new marker for the occurrence of irreversible reactions which needs further investigation. Current amplitudes of 1 *mA* for the considered single electrode configuration did not lead to a capacitive voltage saturation nor a considerable dissolution of platinum ions and is thus considered as a safe operation configuration.

## 1. Introduction

Platinum-iridium (PtIr) electrodes are considered particularly suitable for neural stimulation due to their proven efficiency and stability [1,2]. The topic of safety plays a central role in various applications and is therefore frequently examined. There exist both traditional methodologies and more advanced approaches that depart from laboratory settings to investigate electrochemical systems. Conventional characterization techniques, such as electro impedance spectroscopy (EIS) and cyclic voltammetry (CV), are employed to not only describe the electrochemical system but also determine its safety limits. Therefore, the water window, the reversible potential limits, of platinum-iridium electrodes (−0.6 *V* − +0.8 *V vs*. Ag/AgCl) is defined by performing CV at scan rates of 0.05–0.5 $\frac{V}{s}$ [1,3–6]. However, such scan-rates are not always representative of the real usage scenario, as shown by our investigation. The Charge Storage Capacity (CSC), the maximal reversible charge density at the phase boundary [7], is often consulted to predict reversible limits. But according to Hudak *et al*. the CSC predictions often overestimate the amount of charge that can be injected reversibly during neural stimulation pulsing using platinum microelectrodes [8]. Because the CSC values are recorded at a gradual scan rate during CV, electrochemical reactions have more time to complete before reaching the edge of the water window [8]. Thus, CSC is also not considering real usage scenarios in terms of time-varying pulse shapes applied on the electro-chemical system.

Approaching the current pulse stimulation, transient voltage measurements are performed in order to determine the charge injection capacity (CIC). In this context, the electrochemistry at the electrode-tissue interface is considered when investigating the relationship between charge injection and observed tissue damage [3,9]. Several research investigated the CIC of Pt in vitro, agreeing that 100 to 150$\frac{\mu C}{cm^{2}}$ can be delivered by 200 *μs* pulses [10]. Others have reported a reversible range of 80 to 120$\frac{\mu C}{cm^{2}}$ for the injected charge [11]. Furthermore, Della Valle *et al*. found ultrasmall Platinum-Iridium-Carbon fibre microelectrodes can inject 1.25 $\frac{mC}{cm^{2}}$ applying 0.5 *ms* pulses in PBS while staying in the water window for PtIr [1]. Further investigations with microelectrodes showed that the maximum cathodic excursion at the phase boundary (referred to as *E*_*mc*_) also has a great impact on the evaluation of the reversible boundaries [1,3].

Krishnan *et al*. examined the residual voltage (RV), its origin and growth in microelectrode application in more detail. Looking at the electrode/tissue interface, the RV results from the charging of the capacitive share due to an accumulation of charge across the electrode/electrolyte interface up to a saturation during biphasic current stimulation [4]. Intrinsic RV was also detected in a theoretically perfectly charge-balanced pulse.

The literature presented so far deals with microelectrodes in the application of neurostimulation. There are fewer references and studies on the use of macroelectrodes in neural stimulation. When utilizing macroelectrodes, which are electrodes with a geometric surface area (GSA) exceeding 0.001 *cm*^*2*^ [3], it is possible to provide safe and therapeutic stimulation at recommended charge density limits of 30 $\frac{\mu C}{cm^{2}}$ in the application of DBS [12]. Brummer and Turner conducted a separate study, indicating that Pt macroelectrodes have a maximum charge transferability of 420 to 490 $\frac{\mu C}{cm^{2}}$ while utilizing all reversible charge injection mechanisms [13].

However, the published studies on macroelectrodes in neurostimulation employ still very small electrodes, for instance those utilized in cochlear implants (GSA 2.0 *mm*^2^-2.3 *mm*^2^) [2,14,15], in comparison to the much larger “EASEE” Electrode (Epicranial Application of Stimulation Electrodes for Epilepsy) with GSA 0.9132–3.6528 *cm*^2^. EASEE guided electro stimulation for treating focal epilepsy is designed to stimulate a selected region of the brain below the patient’s perception threshold to counteract epileptic seizures [16,17] and no similar investigation can be found in literature. The novel aspect of this study is first, to provide in-vitro data regarding the use of very large macroelectrodes in Laplace configuration [18,19], with asymmetric low-frequency pulses, higher current amplitudes, and a higher voltage than that observed in microelectrode and smaller macroelectrode applications. Second, we suggest considering a modified set of parameters of traditional characterization techniques and transient voltage measurements to align with the specific requirements of the stimulation application closer to the real usage scenario. This study contributes to existing literature by demonstrating the potential for combining conventional methods with specific stimulation pulse parameters. In this way, an application-related characterization that exceeds conventional electrode characterization is presented.

## 2. Materials and methods

### 2.1. Model

**Fig 1:**
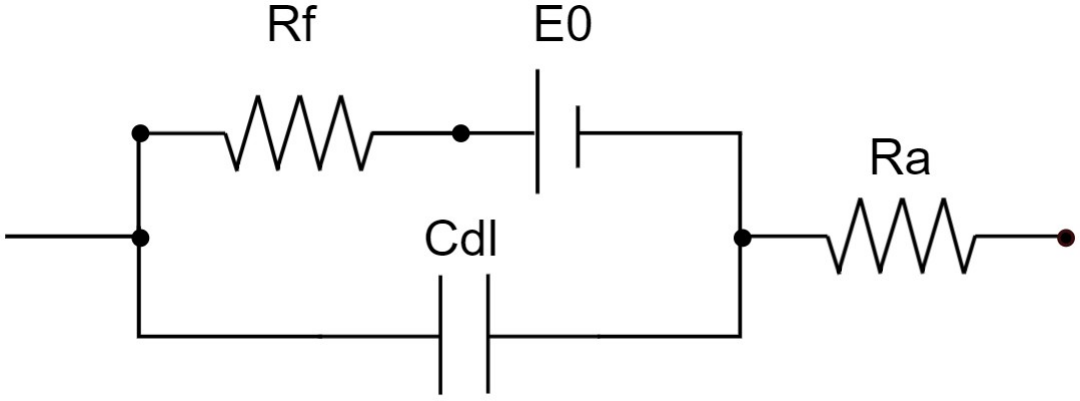
Equivalent circuit diagram of the electrode. *R*_*f*_: Faraday resistor, *E*_0_: Equilibrium potential, *C*_*dl*_: Charging and discharging of the double layer, *R*_*a*_: Resistance of electrolyte. [21].

The equivalent circuit modelling of the electrode in an electrolyte solution, is shown in Fig 1. Given no external current flows, the electrochemical cell is in a state of equilibrium (*E*_0_), resulting in a constant potential across the phase boundary and the electrolyte. When current flows, two charge transfer mechanisms are relevant: non-Faraday reactions and Faraday reactions. Non-Faraday reactions, also known as capacitive charge transfer, involve no exchange of electrons between the electrode and the electrolyte. Instead, the charge is transferred by charging and discharging the Helmholtz double layer at the electrode surface [3,20]. In the model, this is represented by the double layer capacitance *C*_*dl*_. The Faraday resistor *R*_*f*_ models Faraday reactions, including charge transfer reactions occurring at the electrode-electrolyte interface, which can only be represented linearized for low voltages. *R*_*a*_ models the constant resistance of the electrolyte [21]. In this application, there are three electrodes in the electrolyte, a working electrode array (WE) a counter electrode (CE) and the reference electrode (RE) [22].

Faraday processes of reduction and oxidation can transfer electrons between the two phases, creating an electric field. The reversibility of a reaction is determined by its electron transfer kinetics at the interface.

We will further investigate the voltage dropping over C_*dl*_, which is defined as

$$Uc\;(t)=I\;*\;Ra\;*\;(1\;-\;e^{-\frac{t}{Rf*Cdl}})\;$$ (1)

Uc(t) indicates the course of the voltage over time, whereby *E*_*ma*_ and *E*_*mc*_ are the maximum anodal and cathodal driven electrochemical potential excursion [23]:

$$E_{ma}=V_{max}-V_{aa}$$ (2)

$$E_{mc}=V_{min}+V_{ac}$$ (3)

While the maximum voltage (during the anodic phase) and the minimum voltage (during the cathodic phase) are defined as V_max_ and V_min_, respectively, the resistively dropping access voltages across the electrolytic resistance during the anodic and cathodic phases are denoted as V_aa_ and V_ac_.[3].

The frequency dependent impedance course is according to the equivalent circuit diagram defined as:

$$Z=\left(\frac{\;R_{f}}{{1+\omega^{2}R}_{f}^{2}{C_{dl}}^{2}}+\;R_{a}\right)-j\;\frac{{\omega R}_{f}^{2}C_{dl}}{{1+\omega^{2}R}_{f}^{2}C_{dl}^{2}}$$ (4)

### 2.2. Materials

**Fig 2:**
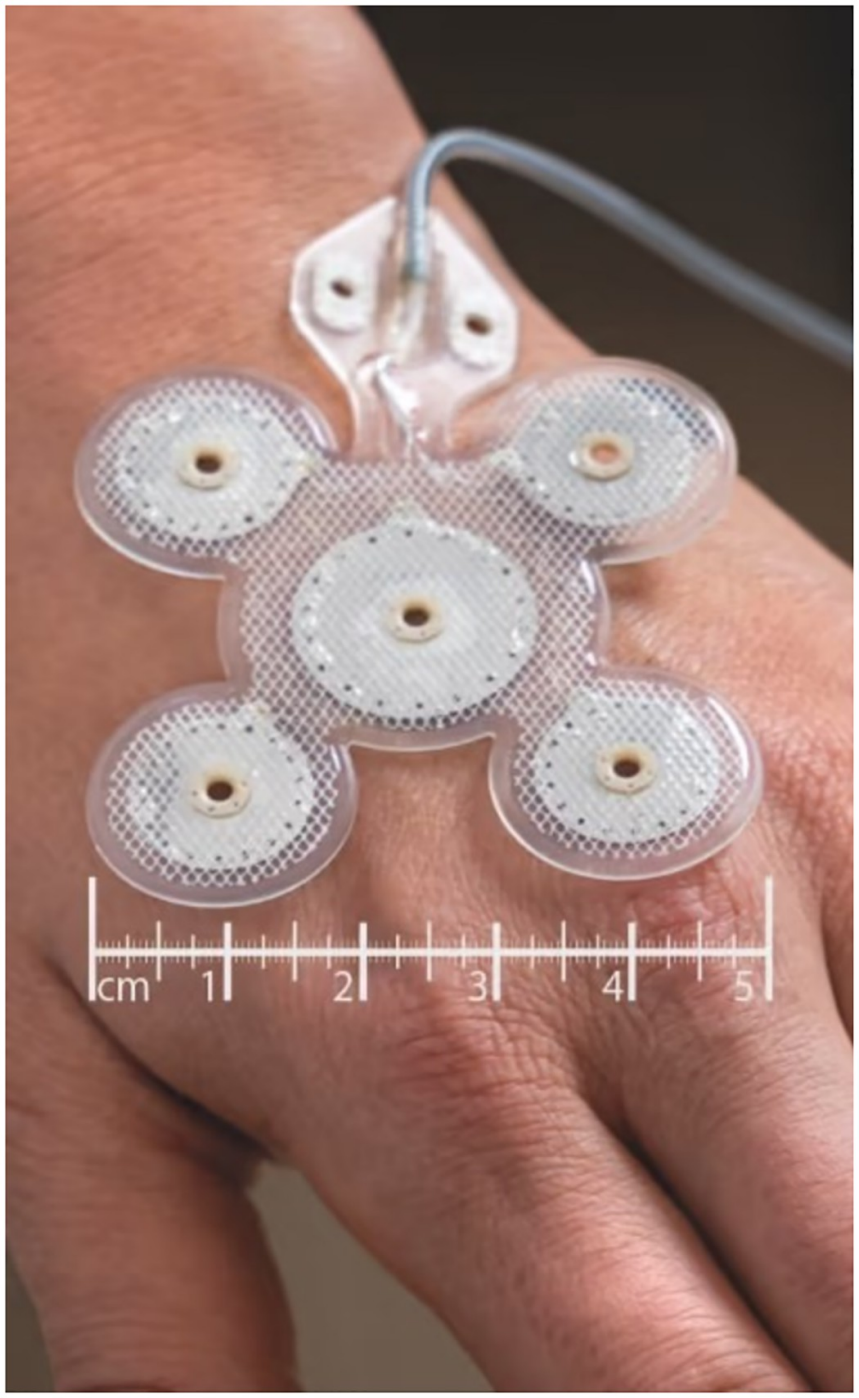
Laplace configuration of EASEE.

All measurements were performed using the Parstat 3000A device by Princeton Applied Research. The Pt-Ir electrode array (90%−10%) developed by Precisis GmbH consists of a larger central counter electrode with a diameter of 15.5 *mm (*GSA 1.7581 *cm*^*2*^*)* and four outer peripheral electrodes, each with a diameter of 10.8 *mm* (GSA 0.9132 *cm*^2^), which are arranged in a Laplace configuration (Fig 2) to achieve a higher level of electric field penetration [15,18,19]. The reference electrode B2920+ from SI Analytics used in this work consists of silver-silver chloride (Ag/AgCl).

The electrolyte solution for the three-electrode configuration is a diluted phosphate buffered saline solution (Gibco^™^ PBS (10x), pH 7.4) with an impedance of 2.225 *k*Ω at 1 *kHz*. This impedance is comparable to the electrode/tissue impedance on the skull. All measurements were performed at room temperature.

### 2.3. Electrochemical impedance spectroscopy

The Electrochemical Impedance Spectroscopy (EIS) was performed by applying a sine wave in a frequency range of 0.1–100 *kHz* and amplitude of 10 *mV* [1,4]. Impedance data was recorded for one electrode and for up to four peripheral electrodes in combination (GSA 0.9132 *cm*^2^–3.6528 *cm*^2^) to analyze the impact of GSA on the electrochemical system. Since the electrode arrangement is symmetrical to the central electrode, the configuration of the active electrodes is irrelevant. What is of interest is how many of the four peripheral electrodes are active. ZView (Version 2.8d) was then used to fit the measurement data to the given impedance model (see Fig 1, Eq 4).

**Fig 3:**
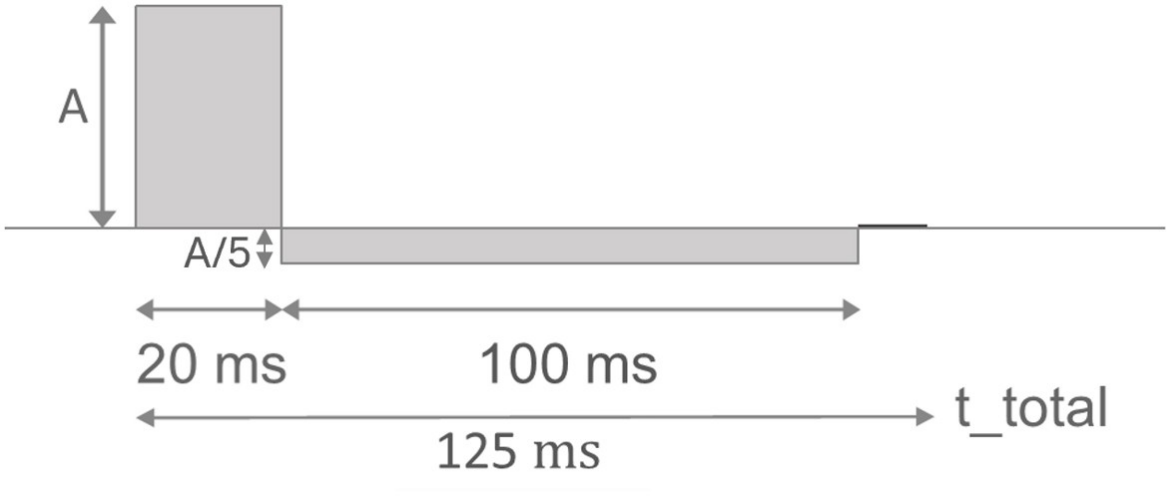
DLS pulse shape. A: Anodic pulse amplitude, t_total: Total pulse width.

Spectral analysis was performed by applying discrete Fourier Transform (DFT) on sampled DC-like stimulation (DLS) pulse data to break down the time-dependent pulse signal into its various frequency components (based on Fig 3, sample rate = 1000 *Hz*, hamming window defined as $W\left(n\right)=0.54-0.46\text{cos}\left(\frac{2\pi n}{M-1}\right);\;0\leq n\leq M-1$, zero padding with 5x signal width before and 5x signal width after signal).

### 2.4. Cyclic voltammetry

The applied potential was varied within specified limits at a defined scan rate. This drives electrochemical reactions at the test electrode and provides information on reversibility and stability [3]. For the CV, the initial focus laid on a single peripheral electrode; in later iterations of the experiment, up to four electrodes were short-circuited in order to study the impact of the geometric surface area (GSA) on electrochemical reactions at the phase boundary. One experiment consisted of 10 cycles. The potential limits were increased from ± 1*V* to ±3 *V* in 1 *V* steps. The scan rate was varied in increments of 1 $\frac{V}{s}$ from 0.1 $\frac{V}{s}$ over 1 $\frac{V}{s}$ to 10 $\frac{V}{s}$. A scan rate of 10 $\frac{V}{s}$ is used to approximate the velocity of the voltage development induced by a current pulse. From the CV data, the CSC of the electrodes was calculated by dividing the observed hysteresis mean area of 10 cycles by the scan rate [3].

To generate more data on reversible limits, we extrapolated the scan rate to a constant, non-variable voltage of -1 V and +1.5 V amplitude for 30 minutes.

### 2.5. Biphasic current pulse test / voltage transient measurements

The peripheral electrode received the anodic pulse of 20 *ms*, followed by the cathodic phase with a pulse width of 100 *ms*. The intervals between the pulses were separated by 5 *ms* of open-circuit potential (Fig 3) [24]. The DLS pulse is a biphasic asymmetric pulse shape, which is used in epilepsy therapy [15,25]. The negative cathodic phase is five times longer than the anodic phase with one fifth of the anodic amplitude, making it charge balanced. In terms of pulse polarity during the stimulation, the central electrode was defined as anode, while the peripheral electrodes were the cathodes [15,26]. Each measurement started at the same initial potential, as the system contained charge for some time after stimulation. To confirm that the remaining voltage was within the valid range of 430 *mV*, close to E_0_, the open circuit potential (OC) was measured. Therefore, the system was stabilized to return to the equilibrium state. The anodic pulse amplitude was increased incrementally from 1 *mA* (cathodic amplitude: -0.2 *mA*) to a maximum of 8 *mA* (cathodic amplitude: -1.6 *mA*) in 1 *mA* intervals, with voltage measurements taken throughout. To visualize further limit values, the experiment was extended with pulse amplitudes 10, 12 and 16 *mA*. In order to achieve a more accurate representation of the data and to minimize the impact of noise in the measured values, a discrete, linear convolution (moving average with a window size of 10 and a weight of 1/10) was employed for data filtering. To relate the scan rate parameter to the current pulses, the first derivative over time was determined from the measured voltages. Furthermore, a capacitive voltage curve fitting procedure was conducted according to Eq 1 at 1 *mA* and 4 *mA* anodic amplitude to ascertain whether a system change had occurred.

### 2.6. Accelerated stimulation protocol

For longer periods of stimulation, the DLS pulse shape was used to obtain 10 hours of continuous stimulation, resulting in 288,000 consecutive pulses. This allowed us to assess the residual voltage (RV) and gain insight into system saturation. All measurements were performed at a anodic amplitude of 1 *mA* and a cathodic amplitude of -0.2 *mA* applied to a single peripheral electrode (GSA 0.9132 *cm*^*2*^). The same PBS solution described in Chapter 2.2 was utilized for all measurements. The anodic pulse widths were then shortened to 15 *ms* and 25 *ms* in order to incorporate a 25% deviation into the pulse shape. The amplitude remained unchanged. The charge per pulse was determined by the real pulse widths recorded by an external data logger. To address concerns regarding reversibility and biocompatibility, platinum analysis was conducted using Inductively Coupled Plasma Mass Spectrometry (ICP-MS) with Limit of Detection (LoD) <0.5 *μg/l* and Level of Discrimination <0.2 *μg/l*.

## 3. Results

### 3.1. Electrochemical impedance spectroscopy

**Fig 4:**
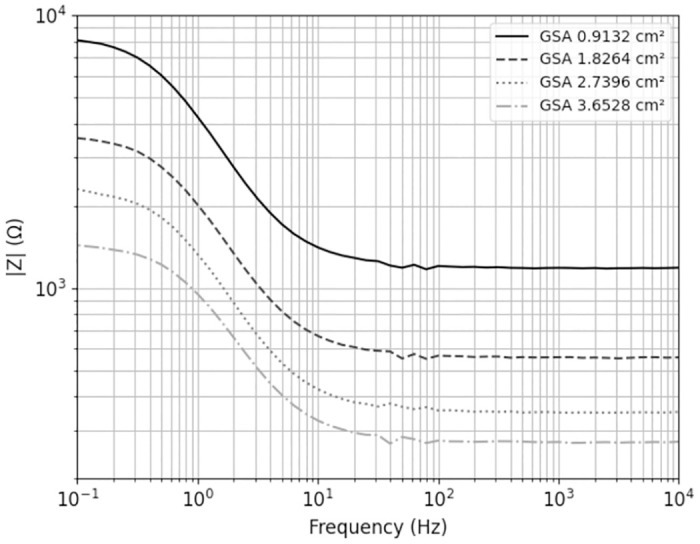
Dependence of different GSA’s of the electrodes in relation to their impedance.

Fig 4 demonstrates the fundamental behavior of the system. By fitting the impedance over frequency, the model can be confirmed, as evidenced by the small error of each component. Further details can be found in Table 1. The graph depicts the RC component’s behavior while also highlighting the impact of the area on impedance magnitude.

**Table 1:** The fitted RC-Model and its components. *R_a_*, *C_dl_* and *R_f_* are the fitted values for each GSA used in the EIS experiments. The Error% is the mean relative error of the measured values to the fitted RC-Model for each of the three components respectively.

GSA (cm^2^)	*R*_*a*_ (*Ω*)	Error% *R*_*a*_	*C*_*dl*_(μF)	Error% *C*_*dl*_	*R*_*f*_(*Ω*)	Error% *R*_*f*_
0.9132	1214	0.94811	35.713	2.6382	6370	2.4328
1.8264	571.3	0.93352	72.315	2.6692	2765	2.3551
2.7396	360.1	1.106	107.4	2.8536	1779	2.5084
3.6528	279.9	1.0105	144.69	3.161	1078	2.534

From 10 *kHz* to 100 *Hz* the electrolyte’s impedance (*R*_*a*_) is displayed. The complex impedance of the capacitance (*C*_*dl*_) is shown for lower frequencies (20 *Hz* to 0.3 *Hz*). The four impedance values converge for frequencies up to 0.1 *Hz* to a limit representing the impedance of the Faraday reactions at the electrode/electrolyte phase boundary together with the electrolyte impedance (*R*_*f*_).

In the electrolyte, with a GSA of 0.9132 *cm*^2^, an impedance of approximately 1.2 *kΩ* was achieved. At 0.1 *Hz*, the impedance measurement is 8.01 *kΩ*, reflecting the combined effects of faradaic reactions and electrolyte resistance.

Spectral analysis of the DLS pulse shape by performing the discrete Fourier transform showed that the main spectral components of the DLS pulse ranged from 8 *Hz t*o 40 *Hz* (Table 2).

**Table 2:** Overview of how much energy lies in what part of the system. 0.1–20 *Hz* capacitive power, 40–10 000 *Hz* resistive power.

Frequency	*8 Hz*	*16 Hz*	*24 Hz*	*32 Hz*	*40 Hz*	*48 Hz*
Normalized spectral energy	100%	86.02%	65.59%	42.18%	19.64%	2.47%

Frequencies with a high proportion of spectral energy are present between 8–16 *Hz*, underscoring the importance of the phase boundary resulting from capacitive charging. More precisely, the EIS results in Fig 4 show the capacitive voltage curve between 1 and 8 Hz. The results of the spectral analysis demonstrate that the energy of the pulse is at 8 Hz, exactly in the range where the EIS results show the capacitive voltage curve. At a chemical level, this is the charging and discharging of the Helmholtz double layer and highlights the importance of the phase boundary.

### 3.2. Cyclic voltammetry

**Fig 5:**
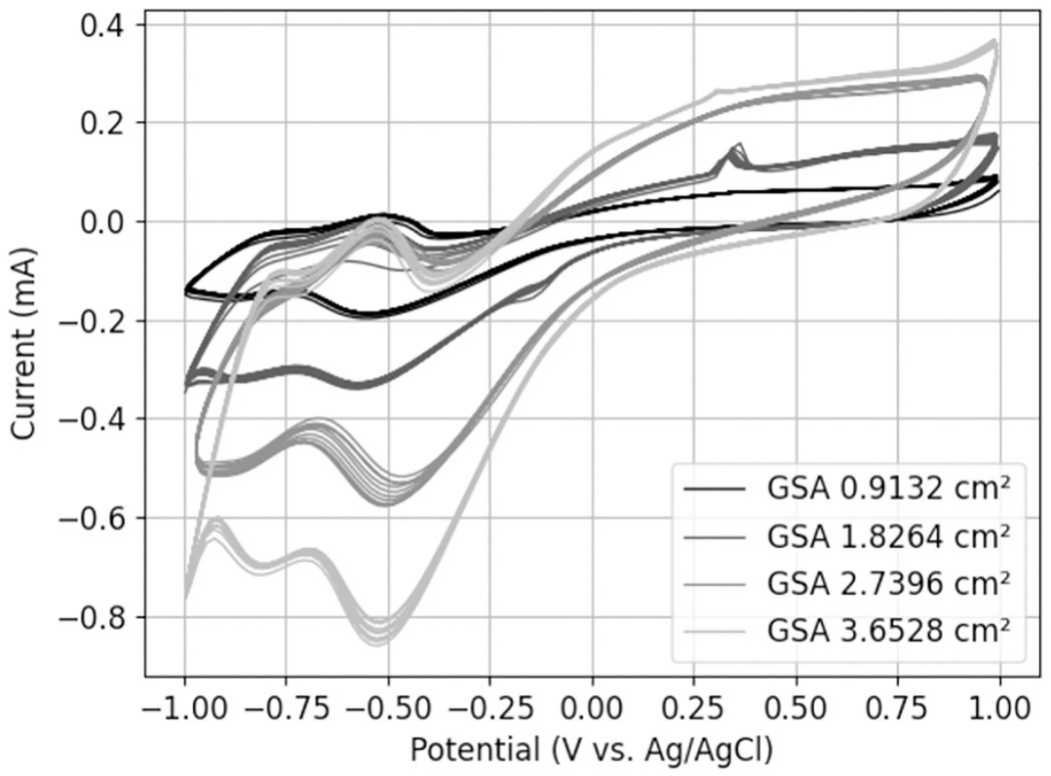
Influence of the electrode area on the cyclic voltammogram. CV was performed with a scan rate of 0.1 $\frac{V}{s}$.

As the electrode area increased, the area of the current-voltage curve widened and more charge could be reversibly transferred across the phase boundary. The cathodic phase processes, i.e. the processes below 0 *A*, were particularly prominent with the increase in the electrode area. Charge density on the other hand remains constant. The wave-like shape suggests electrochemical reactions, as the current fluctuated locally (Fig 5). The reactions may have been reversed upon polarization inversion, as observed by the equivalent wave at -0.5 *V*.

An exponential trend in the waveform is not detected, indicating the presence of reversible processes beyond the limits of the water window. This is why the CSC has been calculated for voltage ranges of up to ± 3 *V* in Table 3 [27]. CSC increases at higher voltage limits. The variations in CSC indicate minor measurement inaccuracies, as it remains constant with GSA.

**Table 3:** CSC determination by means of CV performed with 0.1 $\frac{V}{s}$ scan rate and various voltages ranges.

GSA	0.9132 *cm*^*2*^	1.8264 *cm*^*2*^	2.7396 *cm*^*2*^	3.6528 *cm*^*2*^
Applied voltage ranges	Charge Storage Capacity [*mC/cm*^*2*^*]*			
± 1*V*	1.7733	1.6043	1.8552	1.9654
± 2 *V*	6.30	7.4869	6.3237	8.2769
± 3 *V*	12.7386	15.4859	13.0231	15.1665

**Fig 6:**
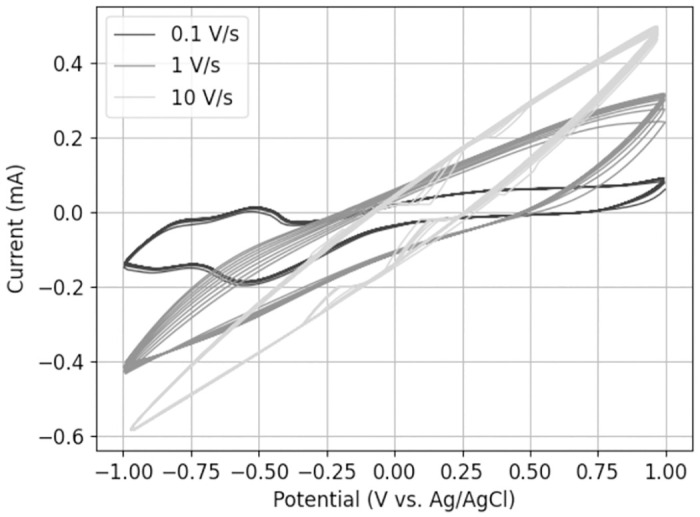
Influence of scan rate on electrochemical system. Performing CV with scan rates of 0.1, 1 and 10 $\frac{V}{s}$.

By moving away from the traditional cyclic voltammetry experiments, we sought to investigate the behavior of the current-voltage curve when the voltage changes at a faster rate. By increasing the scan rate up to 10 $\frac{V}{s}$, less current fluctuation and consequently less electrochemical reaction are observed (Fig 6). The presence of sharp edges in the curve suggests measurement inaccuracies, which may be attributed to the inherent limitations of the measuring device. We discovered gas formation by applying a constant voltage of -1 V, approaching the cathodic threshold, and a voltage of 1.5 V, approaching the anodic threshold, for 30 minutes.

The first derivative of the voltage resulting from the application of one current pulse to the system is of great interest. The first derivative indicates the inclination of the curve, i.e. the “speed of the voltage”. This value can be linked to the applied scan rate of the CV measurements.

**Fig 7:**
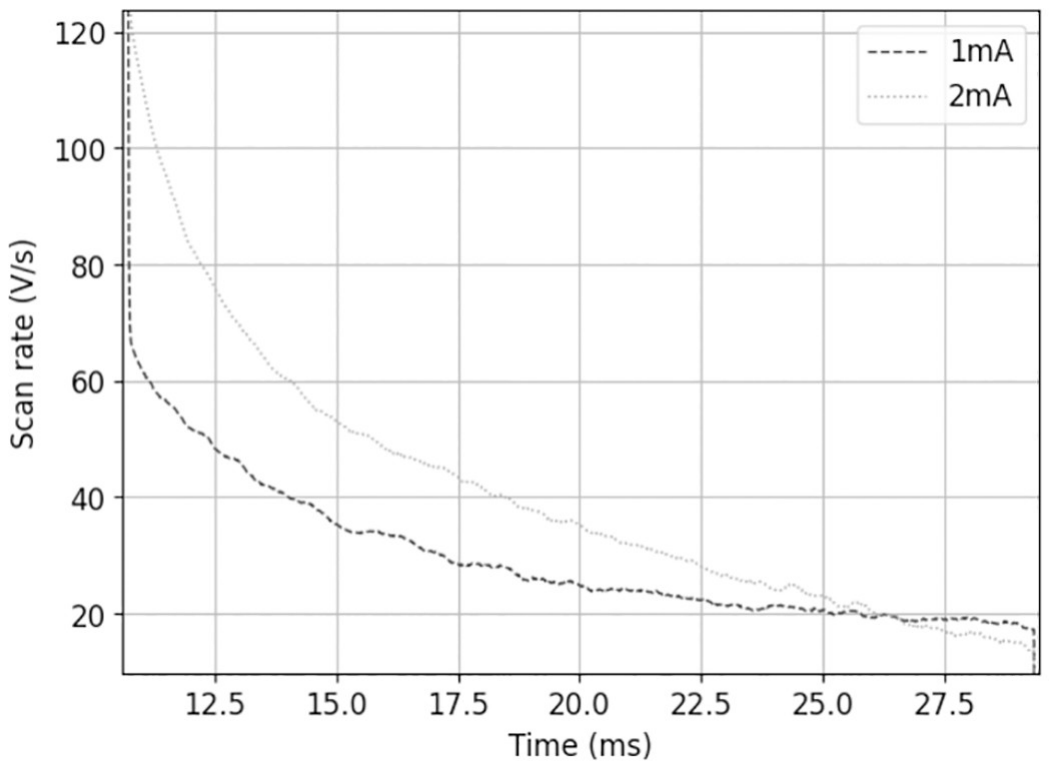
First derivative of transient voltage after current pulse. 1 mA and 2 mA anodal amplitude and pulse width of 20 *ms*; The pulse was applied after 10 *ms* in order to measure the prevailing equilibrium potential.

**Fig 8:**
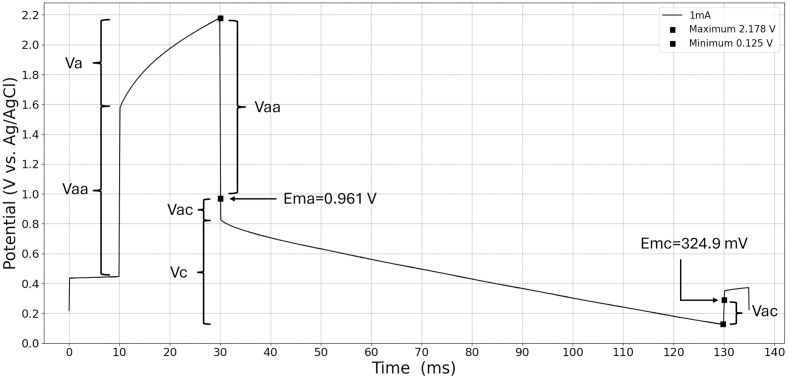
Voltage excursion and current waveforms of 20 *ms* and 100 *ms* pulses in vitro. E_ma_: 0.961 *V*; E_mc_ 324.9 *mV*; pulse amplitude of 1 *mA*.

Thus, the voltage waveform resulting from a DLS current pulse (voltage development for 1 *mA* pulse in Fig 8) was derived over time to display the capacitive voltage change. This was determined for a anodic pulse amplitude of 1 *mA* and 2 *mA* (Fig 7).

The results demonstrate that the voltage change during capacitive charging of the phase boundary in the pulses correlates within a range of 80–20 $\frac{V}{s}$ (excluding the voltage leap of the resistive component), whereas the scan rate in the conventional CV measurements is limited to 0.1 $\frac{V}{s}$. The voltage alteration of the pulse is remarkably higher than the tested scan rates in CV. Therefore, we performed CV measurements with scan rates up to 10 $\frac{V}{s}$ to investigate the effects at different voltage ranges.

### 3.3. Biphasic-current pulse and its significance for capacitive voltages

Significant characteristic potentials, such as maximum anodally driven (*E*_*ma*_) and maximum cathodally driven (*E*_*mc*_) electrode/electrolyte interface excursions, are investigated in detail. These potentials were correlated with the reversible potential limits to estimate the presence of irreversible reactions [9,23]. In Fig 8, the voltage response of a DLS pulse at a anodic amplitude of 1 *mA* is presented.

**Fig 9:**
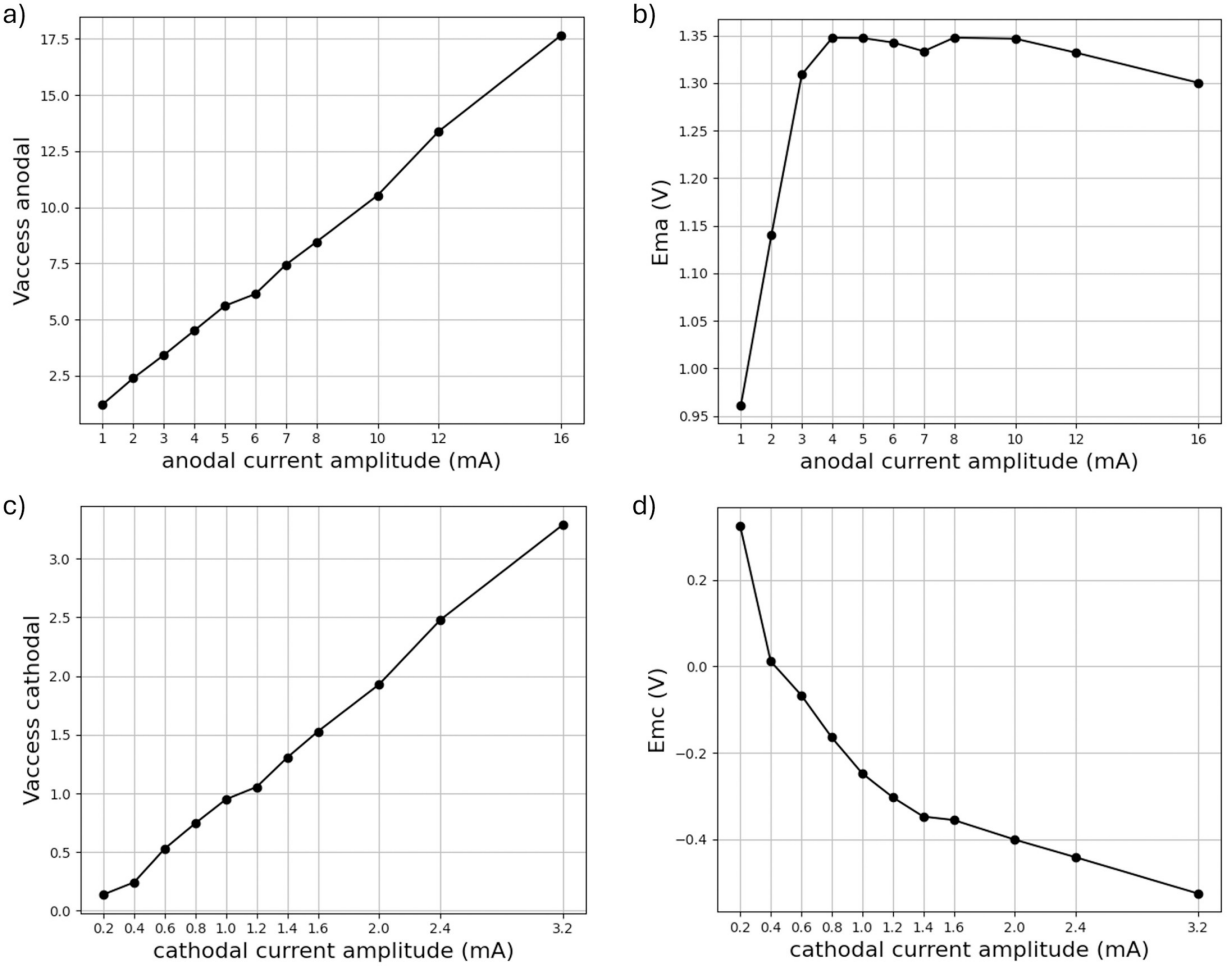
a Access voltage anodal, b Ema, c Access voltage cathodal, d Emc. Characteristic voltage curves as a function of the applied current amplitude. *E_ma_*: Maximum anodally driven electrochemical potential excursion, *E_mc_*: Maximum cathodally driven electrochemical potential excursion, Vaccess anodal (*V_aa_*): Resistive voltage drop across electrolyte resistance in anodal phase, Vaccess cathodal (*V_ac_*): Resistive voltage drop across electrolyte resistance in cathodal phase.

The data set was smoothed with a convolution filter (see section 2.5) before further processing to account for noise. The equilibrium potential of 437 *mV* was recorded 10 seconds prior to the application of the current pulse. This led to the *E*_*ma*_ shifting towards higher ranges at the end of the anodic pulse. The perpendicular voltage rise, i.e. the anodic access voltage (*V*_*aa*_), is the voltage resistively dropped across the electrolyte [9]. The Helmholtz double layer charging and discharging process is defined by the capacitive voltage rise of *V*_*a*_ and *V*_*c*_, defined by Eq 1. *E*_*ma*_ and *E*_*mc*_ are calculated for various current amplitudes of the cathodic DLS pulse in accordance with Eqs 2 and 3, and these results are presented in Table Y of the supplementary material. Fig 9 illustrates the current amplitude’s effect on both *V*_*aa*_ and *V*_*ac*_, as well as the maximum positive and negative polarization at the phase boundary (*E*_*ma*_ and *E*_*mc*_). The access voltage curves in Fig 9a and 9c shows linear behavior in accordance with Ohm’s Law.

Fig 9b presents the *E*_*ma*_‘s evolution, i.e. the voltage at the phase boundary after the anodic pulse, for a variety of pulse amplitudes. Despite an increasing pulse amplitude from 3 *mA* to 16 *mA*, the *E*_*ma*_ stagnates in the range of 1.3–1.35 *V*. In Fig 9d, the *E*_*mc*_ is depicted versus the applied cathodic pulse amplitude. A cathodic pulse lasts five times as long as the anodic pulse and has one fifth of its amplitude to achieve charge balance. Thus, the values shown comprise one experiment, but differ in amplitude and duration. The intensity of the current is a crucial factor and has a higher impact on the voltage at the phase boundary post cathodic pulse.

The varying course of capacitive voltages at different current amplitudes (Fig 9b and 9d) suggests that the model defined in section 2.1 is only valid up to a certain limit. In order to describe the system’s behavior beyond that limit (*E*_*ma*_ exceeding 1.3 V), a different model needs to be established. Therefore, we fitted the curve according to Eq 1 from 4 *mA* to 1 *mA*. This resulted in a root mean squared error value (RMSE) of 0.010 for 4 *mA* with given resistance of 759 Ω and a capacitance of 7.66 *μF*, whereas the error calculated for the same measurement with 1 *mA* and same values was 0.074.

### 3.4. Accelerated stimulation protocol and residual voltage

In order to address the influence of Accelerated Stimulation Protocol and residual voltage development, 10h-measurements (LTM1-LTM4) have been conducted. LTM1 and LTM2 are two identical measurements for repeatability purposes. Table 4 lists the measured charge per pulse, the residual voltage and the dissolved platinum ion concentration of the accelerated stimulation measurements. The charge per pulse was determined from the real pulse widths recorded by an external data logger. The residual voltage is the voltage in the system after exactly 10 hours of stimulation pulses. As indicated by the RV, the voltage range shifts away from the equilibrium potential to negative or positive ranges depending on the charge imbalance per pulse.

**Table 4:** Overview of accelerated stimulation protocol and setup. Charge per pulse, residual voltage determination and platinum concentration using ICP-MS with Limit of Detection (LoD) <0.5 *μg/l* and Level of Discrimination <0.2 *μg/l*.

Accelerated stimulation protocol (LTM)				Charge disbalance per pulse*	Residual voltage	Platinum concentration
LTM 1	anodic	cathodic	OC	-1.25 μC (cathodic charge)	-1.14 V	0.52 μg/l **
real	18.8 ms	100.0 ms	6.2 ms			
LTM 2				-1.24 μC (cathodic charge)	-1.19 V	0.42 μg/l **
real	18.8 ms	100.0 ms	6.2 ms			
LTM 3				-5.0 μC (cathodic charge)	-2.87 V	1.0μg/l
real	15.0 ms	100.0 ms	5.0 ms			
LTM 4				+3.7 μC (anodic charge)	1.53 V	4.2 μg/l
real	23.8 ms	100.0 ms	6.2 ms			

*Charge disbalance value was calculated based on parallel voltage monitoring values.

** within range of LoD.

Despite a larger shift of the residual voltage from the equilibrium potential to -2.865 *V* (LTM 3), fewer platinum particles were detected in the solution compared to the positive shift of the residual voltage to 1.53 *V* (LTM 4).

## 4. Discussion

### 4.1. Significance of the spectrum

The good fit of the impedance curve of the extremely large electrodes confirms the model due to the small relative error. In addition, we establish an application-specific relationship between the EIS results and the spectral analysis of the DLS pulse. Linking the spectral analysis data to the results obtained from EIS reinforces the significance of the maximum positive and negative polarization at the phase boundary.

In our DLS experiments, the *E*_*ma*_ dominates over *E*_*mc*_ due to the substantially higher anodic amplitude. After analyzing the pulse’s spectrum and linking the EIS results to the frequency components of the DLS pulse, we observed a strong correlation between the effective frequency of the capacity and the frequency emitted in the pulse (the highest frequency composition according to DFT). Since most of the energy is effectively transmitted to the model’s capacitance when pulses are applied, crucial reactions occur among the phase boundary, as *C*_*dl*_ represents the phase boundary in the model (Fig 1). Therefore, we use the *E*_*ma*_ data to make statements about the reversibility of the system, which is further discussed in Chapter 4.3. Consequently, the Helmholtz double layer, which forms at the phase boundary, is of great importance, as we have confirmed that the spectral energy lies there.

### 4.2. Significance for the reversible boundaries

After observing gas formation by applying constant voltages of -1 V and +1.5 V, i.e. scan rate = 0, the reversible boundaries were certainly exceeded, as gas formation is irreversible. However, by extrapolating the results towards higher scan rates, approaching those of DLS pulsing, no gas bubbles were seen despite similar voltage levels. Therefore, we can conclude that the speed at which the voltage in the system changes is an important factor in determining its reversible limits. Linking this finding to the transient voltage measurement results we can show that less electrochemical processes are triggered at higher scan rates. Consequently, we assume less irreversible reactions at high scan rates of 20 $\frac{V}{s}$ to 80 $\frac{V}{s}$. These scan rates correspond to the 1 *mA* anodic amplitude measurement (for GSA = 0.9132 *cm*^*2*^) and implying safe stimulation pulses. Presumably, Faradaic and non-Faradaic reactions are dependent on the voltage scan rate. According to Hudak et al., this is the rationale for CSC overestimating the actual charge reversibly stored at the phase boundary. This occurs because the CSC is derived from CV using a low scan rate [8]. The electrochemical reactions of the ions may be triggered to given potentials (meaning reversible potential limits/water window) but do not evolve into irreversible reactions. Thus, due to their specific reaction kinematics, the ions may not react before the cathodic phase reverses the initiated reaction. This is also evident in the CV experiments whose reversal potentials lie in the water window. Current fluctuations occur and are subsequently reversed in the cathodic phase.

### 4.3. Significance of capacitive voltages

We clearly observed an *E*_*ma*_ stagnation with increasing current pulse amplitudes (Fig 9b). The varying course of capacitive voltages at different current amplitudes (Fig 9b and 9d) suggests a modification in the model. The fitting procedure revealed this model change between 1 *mA* and 4 *mA* anodic current pulse amplitude for one electrode pair. According to Hudak et al. this may be a result of transferring charge into different electrochemical processes [28]. Additionally, it indicated a higher affinity for the occurrence of irreversible reactions at current amplitudes of 4 mA and above. Cogan et al. found that the capacitive potential for reversible charge transfer must lie in the water window [23]. Our DLS pulse measurements across a range of anodic amperages showed no sign of irreversible reactions until *E*_*ma*_ reached a plateau of 1.3 to 1.35 *V* for currents above 3 *mA*. We propose that irreversible reactions occur at this point onwards extending the water window to approximately 1.3 *V* due to faster scan rates. The hypothesis was confirmed by the platinum analysis results during the LTM experiments. However, these pulses are not currently being employed in therapeutic settings. However, if they are to be used therapeutically, their effects must be investigated.

From an electrochemical perspective, the current in our system is generated through ion shifts, resulting in the charging of the Helmholtz layer, and ion movements across the interface, known as Faraday reactions. Once the water window’s limit is reached, a new current path is created due to the occurrence of hydrolysis. This new pathway can lead to a decrease in Faraday resistance as the current increases, thus inhibiting any further increase in voltage. This effect was observed in Fig 9b and 9d. Despite the application of higher current amplitudes, the voltage remained at a constant level. It is proposed that this stagnation is the result of irreversible reactions. The data indicates that when DLS amplitudes exceed 3 *mA*, it is probable that the water window will be exceeded, thereby rendering safe stimulation non-guaranteed.

### 4.4. Accelerated stimulation protocol

The RV correlates with the charge imbalance applied to the system. Krishnan et al. described the RV as a characteristic of the electrode tissue interface and therefore use it for damage detection. This is because charge mismatch is a systematic error due to electronic inefficiencies [4]. We have also taken up this charge mismatch, artificially amplified it and also investigated whether a strong charge imbalance has an effect on platinum dissolution. A negative RV due to cathodic charge overshoot causes the entire system to shift towards negative voltage ranges. With an anodic overcharge, the RV shifts to increasingly positive regions. In order to estimate the severity of RV with regard to safe stimulation, the platinum determination in the solution is taken into account. The results indicate an anodic charge excess having a greater impact on the dissolution of platinum ions than a cathodic charge excess. Despite a more negative RV, less platinum was dissolved. This is possibly explained by five times greater anodic current amplitude, which allows 4.2 times more platinum ions to detect in solution.

According to the daily intake dose limit for platinum ions, the Permitted Daily Exposure (PDE), from the International Council for Harmonisation of Technical Requirements for Pharmaceuticals for Human Use the permissible, parenteral value is 10.8 *μg/day* [29]. The Accelerated Stimulation Protocol of DLS is not representative for a statement in the patient, but only corresponds to a dose that would generally be applied to a patient under differing physical conditions in 30 days(20 min per day). For LTM3, 1 *μg/l* platinum ions were detected in a 60 *ml* solution after 10 hours of stimulation, resulting in a daily delivery of platinum dose of 33.3 *ng/l per day*, which is significantly less than 10.8 *μg/day*.

The platinum exposure from the experiment with a positive residual voltage falls well within the acceptable limits with a release of 140 *ng/day*.

LTM 1 and 2, with 17.3 *ng/day* and 14 *ng/day*, respectively, are considered a safe stimulation range. The charge-balanced 1 *mA* single pulse measurement with an *E*_*ma*_ value of 0.96 *V* corresponds to the platinum ions detected in LTM 1 and 2 of 17.3 and 14 *ng/day*.

## 5. Conclusions

By characterizing the electrodes operating in the system and discussing the stimulation pulses, the electrode-electrolyte phase boundary has been given particular importance. The Fourier spectrum of applied DLS pulses reinforces this importance. Thus, electrochemical processes at the phase boundary may increase at higher anodic current amplitudes and result in irreversibility. Focusing on the capacitive voltage drop, we observed a relationship between the scan rate and the intensity of the current fluctuations. The capacitive voltage induced by the current pulse (1 *mA* anodic pulse amplitude) changes to up to 80 $\frac{V}{s}$. This results in much faster scan rates than those used for traditional cyclic voltammetry to determine the specific character of the electrochemical reactions. We assume less electrochemical reactions occur at a faster scan rate because the polarity of the system is quickly reversed, underlined by very low dissolved platinum concentrations observed during accelerated repetitive stimulation of DLS pulses (LTM1 and LTM2). By demonstrating a model change between 1 *mA* and 4 *mA* pulse amplitude, we presume little indication of irreversible reactions occurring at lower current amplitudes. The LTMs have confirmed this by demonstrating safe operation with 10–20 *ng/day* for 1 *mA* anodic amplitude DLS pulses, which is significantly less than PDE. Even in the highly imbalanced scenario, 100 times less platinum is dissolved than specified in the PDE. Moreover, the *E*_*ma*_ curve appears to reach a plateau at current pulse amplitudes exceeding 3 *mA*, implying an abandonment of reversible boundaries. Potentially, an incidental current of ions could occur in the system, indicating hydrolysis, which is clearly irreversible. Considering the main results, safe and effective stimulation at the operating level of 1 *mA* can be ensured. The Pt concentration determined in the laboratory also provides further proof of biocompatibility.

## Supporting information

S1 File Accelerated stimulation protocol data. (TXT)

S2 File CV scanrate data. (TXT)

S3 File CSC data. (TXT)

S4 File EIS data. (TXT)

S5 File Transient voltage measurement data DLS pulse experiment 1 mA– 6 mA. (TXT)

S6 File Transient voltage measurement data DLS pulse experiment 7 mA– 16 mA. (TXT)

S7 File Table Y overview of asymmetric DLS pulse experiment. (PDF)
