# Supplementary material for: Quantification of safe operation conditions for large-area platinum-iridium electrodes in neurostimulation application
Source: PLoS One. 2024 Dec 19;19(12):e0315779. doi: 10.1371/journal.pone.0315779 (PMC11658615; doi:10.1371/journal.pone.0315779)
Supplement: S7 File — (PDF) [file pone.0315779.s007.pdf]

Experiment ID	Anodic current [mA]	Cathodic current [mA]	V_{aa} [V]	V_{ac} [V]	E_{ma} [V]	E_{mc} [V]
1	1	-0.2	1.216	0.139	0.961	0.325
2	2	-0.4	2.384	0.0243	1.139	0.012
3	3	-0.6	3.413	0.530	1.309	-0.066
4	4	-0.8	4.506	0.746	1.347	-0.164
5	5	-1	5.611	0.950	1.347	-0.247
6	6	-1.2	6.139	1.055	1.342	-0.303
7	7	-1.4	7.4446	1.306	1.333	-0.347
8	8	-1.6	8.460	1.528	1.347	-0.355
9	10	-2	10.531	1.929	1.346	-0.401
10	12	-2.4	13.383	2.479	1.331	-0.442
11	16	-3.2	17.652	3.289	1.300	-0.525
